# Male Accessory Gland Protein Reduces Egg Laying in a Simultaneous Hermaphrodite

**DOI:** 10.1371/journal.pone.0010117

**Published:** 2010-04-12

**Authors:** Joris M. Koene, Wiebe Sloot, Kora Montagne-Wajer, Scott F. Cummins, Bernard M. Degnan, John S. Smith, Gregg T. Nagle, Andries ter Maat

**Affiliations:** 1 Animal Ecology, Faculty of Earth and Life Sciences, VU University, Amsterdam, The Netherlands; 2 School of Biological Sciences, University of Queensland, St. Lucia, Queensland, Australia; 3 Biomolecular Resource Facility, University of Texas Medical Branch, Galveston, Texas, United States of America; 4 Department of Cellular Biology and Anatomy, Medical College of Georgia-University of Georgia Medical Partnership Campus, Athens, Georgia, United States of America; 5 Max-Planck-Institute for Ornithology, Behavioural Neurobiology, Seewiesen, Germany; Temasek Life Sciences Laboratory, Singapore

## Abstract

Seminal fluid is an important part of the ejaculate of internally fertilizing animals. This fluid contains substances that nourish and activate sperm for successful fertilization. Additionally, it contains components that influence female physiology to further enhance fertilization success of the sperm donor, possibly beyond the recipient's optimum. Although evidence for such substances abounds, few studies have unraveled their identities, and focus has been exclusively on separate-sex species. We present the first detailed study into the seminal fluid composition of a hermaphrodite (*Lymnaea stagnalis*). Eight novel peptides and proteins were identified from the seminal-fluid-producing prostate gland and tested for effects on oviposition, hatching and consumption. The gene for the protein found to suppress egg mass production, Ovipostatin, was sequenced, thereby providing the first fully-characterized seminal fluid substance in a simultaneous hermaphrodite. Thus, seminal fluid peptides and proteins have evolved and can play a crucial role in sexual selection even when the sexes are combined.

## Introduction

In internally fertilizing animals sperm are usually accompanied by seminal fluid, forming the ejaculate. Within an ejaculate, natural selection favors substances that activate and nourish sperm and that are thus essential for proper fertilization of oocytes [1]. Additionally, post-copulatory sexual selection favors the evolution of seminal substances that further enhance fertilization chances by influencing female physiology [2], which thereby fall within the definition of allohormones [3]. Selection for such substances is strongly enhanced when sperm recipients store sperm, mate promiscuously and have specialized sperm-digesting organs, because sperm donors that manipulate these processes to the advantage of their sperm's fertilization chances will have a clear evolutionary advantage. Indirect evidence for the presence of such substances is abundant, but very few studies have attempted to unravel their identities. Moreover, although focus has been almost exclusively on separate sex (dioecious/gonochoric) species, we demonstrate here that such substances have also evolved in simultaneous hermaphrodites.

By far the best studied system in separate sex species, in which seminal peptides provide clear reproductive fitness advantages to males, is the fruit fly *Drosophila melanogaster*. Approximately 20 accessory gland proteins (Acps) have been identified and numerous other proteins have been shown to be transferred along with the sperm [4], [5]. These peptides affect females in several ways [6], [7]. For example, Acp70A (also called sex peptide) decreases female receptivity and increases egg production [8]. Furthermore, Rice [9] showed, using an experimental evolution approach, that more competitive ejaculates can be detrimental to the survival of females. This reduced survival is most likely caused by Acp62F, which seems to protect sperm within the female tract but at the same time has a toxic effect on the female [10]. Recently, an additional example of an identified substance promoting sperm storage in the female emerged from analyses of the mating plug of the mosquito *Anopheles gambiae* [11]. There are also several detailed studies into the composition of seminal fluids, e.g., the honey bee *Apis mellifera* [12] and the sea urchin *Lytechinus variegatus* [13] but these have not included functional tests.

In simultaneous hermaphrodites, the best known example of the transfer of an accessory gland substance is probably the shooting of so-called love-darts in land snails [2], [14]. This substance is not transferred along with the sperm, but rather by injection through the partner's skin using a calcareous dart loaded with the substance. This introduced allohormone increases the shooter's paternity [15], probably via the inhibition of sperm digestion [16]. There are similarly idiosyncratic behaviors achieving analogous goals in other hermaphrodites [e.g. 17], [ earthworms; 18, sea slugs] as well as in separate sex species [e.g. 19, salamanders].

Strikingly, the transfer of substances via the seminal fluid has not been convincingly demonstrated in simultaneous hermaphrodites, even though this seems the method of choice in species with separate sexes. The existing theoretical framework predicts a vital role for sexual selection in simultaneous hermaphrodites despite the fact that the sexes are joined within each individual [20]–[23], and there is reason to believe that substances transferred by the ejaculate have an important role to play.

Recent evidence demonstrates the existence of sexual selection in hermaphroditic organisms [2], [15], [24], even though Darwin initially did not envision this [25]. In order to test the presence and the function of seminal fluid substances, we used the hermaphroditic pond snail *Lymnaea stagnalis* (L.). We already know that in this species there is a clear scope for sperm competition. These animals donate and receive sperm frequently, and their egg masses usually contain eggs fathered by multiple partners [26]. In addition, although donors can donate sperm repeatedly, they prefer to inseminate novel partners each time [27] and they transfer more sperm to virgins than to previously-inseminated partners [28], which is in accordance with sperm competition theory [29], [30]. Most importantly, there are clear indications that as yet unidentified seminal peptides and/or proteins, produced by the prostate gland, are transferred to the partner that affect egg laying behavior [31]–[34].

## Results

### Purification and characterization of *Lymnaea* prostate gland peptides and proteins

**Figure 1:**
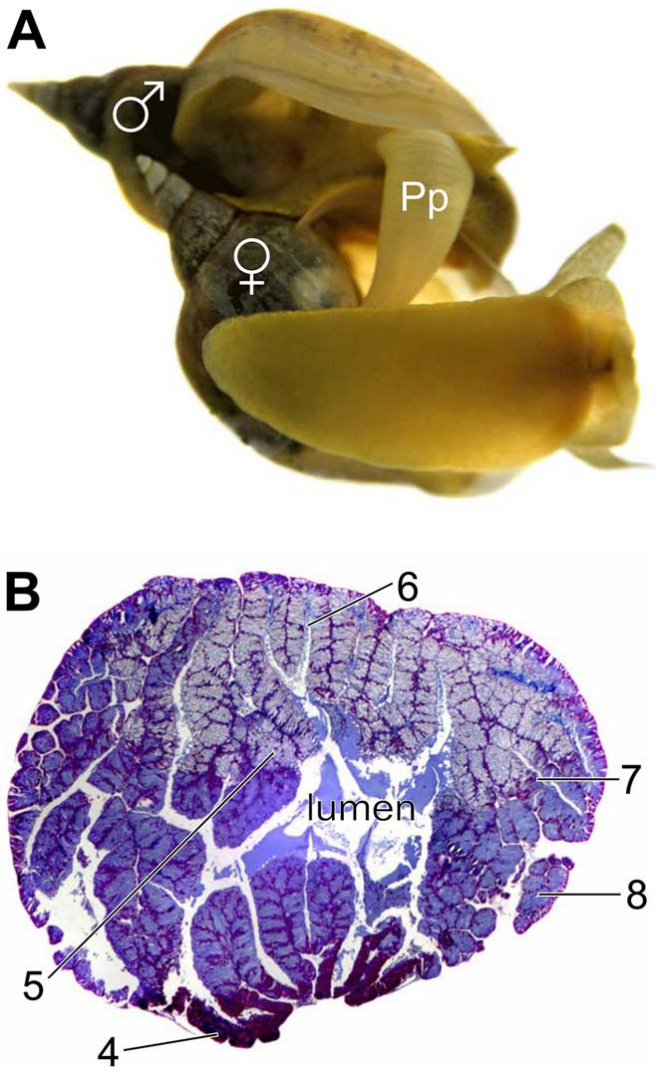
Semen transfer in the hermaphroditic pond snail *Lymnaea stagnalis*. **A.** The photo illustrates the typical mating position of this species. The top snail is performing the male role (sperm donor), its white preputium (penis-carrying organ, Pp) can be seen inserted under the shell of the sperm recipient, where the female opening is located. During insemination, sperm (from the seminal vesicles) and seminal fluids (from the prostate gland) are transferred. Since these are simultaneous hermaphrodites, sexual roles can be swapped immediately afterwards. **B.** Histological section of a prostate gland from *L. stagnalis*. The section illustrates the 5 different types of secretory cells present (numbered type 4 to 8; types 1 to 3 occur in the sperm duct) [43] as well as the presence of secretions in the gland's central lumen through which the sperm pass on their way to the sperm recipient. This 7 µm section was stained with azan as well as hematoxylin and eosin.

**Figure 2:**
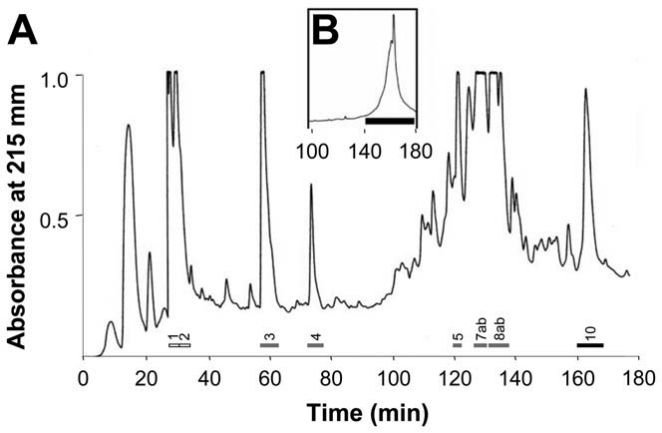
Purification of *Lymnaea stagnalis* seminal fluid peptides and proteins. **A.** C18 RP-HPLC profile of an extract of approximately 40 *Lymnaea* prostate glands that was purified on a Sep-Pak Vac cartridge and fractionated using a gradient of 0.1% HFBA and 100% ACN/0.1% HFBA. Subsequent microsequence analyses confirmed the presence of N-terminal sequences corresponding to the prominent peaks labeled with solid bars and numbers, while the white bars revealed no peptide presence. **B.** The inset shows the repurification of fraction 10 (Ovipostatin) using a gradient of 0.1% TFA and 100% ACN/0.1% TFA. Fractions containing Ovipostatin are indicated by the solid black bar.

In *Lymnaea stagnalis*, upon insemination the sperm donor transfers semen, containing sperm and prostate gland products to the receiving partner (Fig. 1A). Histological sections of the prostate gland reveal several different glandular cell types that release their products into the gland's central lumen (Fig. 1B). Therefore, at least several different seminal fluid components are expected to be transferred along with the sperm. In order to identify some of these components, we prepared protein extracts from prostate glands and separated them using reverse phase-high performance liquid chromatography (RP-HPLC). A representative RP-HPLC elution profile is shown in Fig. 2A. Ten major absorbance peaks were further purified by RP-HPLC using a different counterion. This is shown for the bioactive fraction, see below, corresponding to peak 10 (Fig. 2B). From this peak a 31-residue N-terminal partial sequence was obtained by microsequence analysis that did not match any sequences in GenBank or PIR databases. Table S1 shows the amino acid sequences of the peptides and proteins found in the major absorbance peaks that contained peptides or proteins (see also Fig. 2A), none of which show any resemblance with known sequences in the abovementioned databases.

### Peptide and protein tests

**Figure 3:**
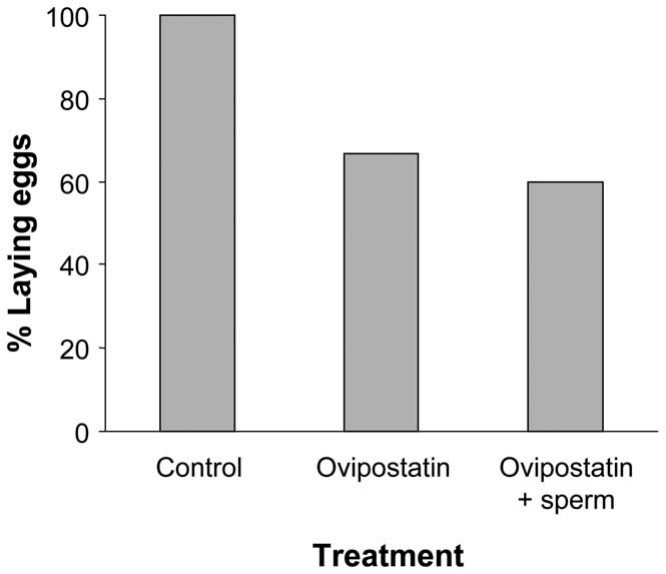
Effect of Ovipostatin on egg mass production of *Lymnaea stagnalis*. The graph shows the percentage of animals laying eggs after intravaginal injection of either the control substance (saline), Ovipostatin, or Ovipostatin accompanied with sperm. N = 15 for each treatment; see text for details and statistics.

We tested the newly-identified peptides and proteins in a bioassay that was performed as follows. Individual snails were injected intravaginally with a peptide or protein at a biologically relevant concentration. The injected solution was tested with and without sperm. Animals were randomly assigned to treatment groups (see methods for details). As shown in Table 1, most of the peptides and proteins tested did not have a significant effect on body size, egg mass production, hatching success or food consumption. The experiment where peptide peak 5 and protein peak 10, both with and without the presence of sperm, were compared with control injection showed a significant effect. Subsequent testing indicated that this effect was due to a significant reduction in the number of animals producing egg masses in the peak 10 treated animals, as illustrated in Fig. 3 (Pearson *χ^2^* = 7.46, *df* = 2, *P* = 0.024). In this part of the experiment the controls laid 1.13±0.35 egg masses on average (N = 15), the peak 5 treated animals without sperm laid 1.25±0.68 egg masses (N = 16) and those with sperm 1.13±0.74 (N = 15), whereas the peak 10 treated animals, laid 0.73±0.59 (N = 15) and 0.67±0.62 (N = 16) egg masses, respectively (see Table S2 for details of all tests). In addition, the average number of eggs was also reduced by peak 10 (ANOVA: *F_2,42_* = 3.77, *P* = 0.03; Post-hoc: control was significantly different from both test solutions, *P*<0.05). The average number of eggs laid by the controls was 103.7±30.8 (N = 15) versus, respectively, 62.67±58.3 (N = 15) and 58.47±55.8 (N = 15) for protein 10 with and without sperm. Hence, protein 10, which we named Ovipostatin, caused its effect irrespective of the presence of sperm. That sperm alone had no effect on egg mass production was confirmed by the control experiment, in which sperm or heptafluorobutyric acid alone did not alter oviposition. These findings agree with previous tests in which the complete seminal fluid was found to suppress egg laying [34].

**Table 1:** Effects of eight *Lymnaea stagnalis* seminal fluid peptide and protein peaks.

Treatments	Body size				Egg mass production			Hatching				Consumption		
	*X^2^*	*F*	*df*	*P*	*X^2^*	*df*	*P*	*X^2^*	*F*	*df*	*P*	*X^2^*	*df*	*P*
Peak 3± spermPeak 4± spermControl	2.26		4	0.69	7.75	4	0.10		0.47	4, 41	0.76	3.90	4	0.42
Peak 7a± spermPeak 7b± spermControl	4.75		4	0.31	7.73	4	0.95	2.60		4	0.63	2.44	4	0.65
Peak 8a± spermPeak 8b± spermControl		0.60	4, 68	0.66	7.22	4	0.12	3.95		4	0.41	3.53	4	0.47
Peak 5± spermPeak 10± spermControl	3.58		4	0.47	10.16	4	**0.04**		0.61	4, 57	0.66	1.85	4	0.76
HFBASpermControl		0.57	2, 54	0.58	4.11	2	0.13	0.74		2	0.69	0.93	2	0.63

The effects of each separate peptide or protein were tested on body size (shell length in mm), egg mass production (total number of masses laid), hatching success (percentage of hatched eggs) and consumption (in cm^2^ lettuce). The five rows represent separate experiments done at different dates. For brevity, the plus-minus sign (±) is used to indicate that the peptide/protein peaks were each tested both with and without added sperm. For parametric tests (ANOVA) the two degrees of freedom (*df*) and *F*-values are shown. For non-parametric tests (Kruskal Wallis) one *df and X^2^*-approximations are shown. Significant differences are indicated in bold. Ovipostatin corresponds to Peak number 10.

### Ovipostatin cDNA and tissue-specific reverse transcription-polymerase chain reaction (RT-PCR)

**Figure 4:**
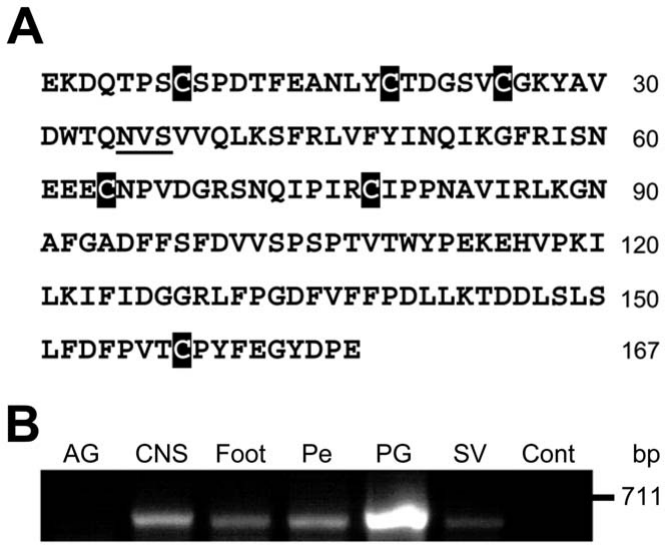
*Lymnaea stagnalis* prostate gland Ovipostatin gene analysis. **A.** Amino acid sequence of prostate gland Ovipostatin. The cDNA encodes a precursor protein of 167 amino acids. A putative glycosylation site is underlined and cysteines are in black shading. The predicted molecular mass of Ovipostatin is 18.9 kDa. **B.** Tissue expression of Ovipostatin mRNA in *L. stagnalis*. Transcript specific primers were used in RT-PCR to identify expression. Ovipostatin mRNA is present (631 bp) in the pooled central nervous system (CNS), foot tissue (Foot), penial complex (Pe), prostate gland (PG) and seminal vesicle (SV). No Ovipostatin mRNA was detected in the albumen gland (AG) or the control in which no cDNA template was used in PCR.

For the protein that showed bioactivity in our tests, we went on to sequence the gene. The prostate gland Ovipostatin cDNA was identified (690 base pairs) and shown to encode a 167-residue precursor (Fig. 4A). The 167-residue mature protein contains 6 Cys residues and a single predicted N-glycosylation site (N_35_VS) (Fig. 4A). The nucleotide sequence of Ovipostatin has been submitted to the GenBank database with accession number GQ906707. The tissue expression of Ovipostatin mRNA was examined by RT-PCR analysis (Fig. 4B). As expected, Ovipostatin mRNA was present (631 bp) in the prostate gland. Ovipostatin mRNA was also detected in the central nervous system, foot tissue, penial complex and seminal vesicle. No amplicon was observed from albumen gland cDNA or the negative control.

## Discussion

Sexual selection favors the transfer of substances, called allohormones, that enhance the reproductive success of the sperm donor [2], [3], [35]. While there is abundant evidence for the effects that seminal fluid peptides and proteins can have on females (in animals with separate sexes), we demonstrate for the first time that such peptides and proteins are also transferred by hermaphroditic animals.

The reported peptide, Ovipostatin, is produced in the prostate gland and transferred during mating, in the ejaculate, along with the sperm. The bioactive factor from the prostate gland was localized to a prominent HPLC peak, which was repurified to homogeneity. N-terminal microsequence analyses of the purified fraction demonstrated that it contained a single N-terminal partial 31-residue sequence. The remaining sequence was obtained by 3′- and 5′-RACE, which resulted in the complete cDNA sequence of Ovipostatin. This protein turns out to be a novel substance, since its predicted sequence showed no resemblance with any protein identified to date.

The finding that Ovipostatin reduces egg mass production in the recipient corroborates earlier work indicating that substances in the semen may influence egg laying [31], [32], [34]. By transferring biologically relevant amounts, our data convincingly demonstrate that it is a seminal fluid protein, and not the sperm themselves, that mediates the observed reduction in egg laying. Besides the inhibition of egg laying, we find that Ovipostatin does not affect hatching success of the eggs or lettuce consumption by the recipient.

Since the reduction in egg mass production in itself seems maladaptive, we expect that Ovipostatin (or one of the other prostate gland components) has another fitness enhancing function (of which reduced egg laying may be a side effect). There are several possible scenarios that could provide an explanation here. First, the observed effect may represent a general inhibition of the female function that also inhibits the willingness to further mate. This could function to prevent additional matings with other partners and/or to allow time for the donated sperm to reach the storage site. Although this would provide a direct benefit for the sperm donor, remating in the female role seems not to be inhibited given that sperm recipients are often inseminated several times in a row by different partners [26], [27].

Second, inhibited egg laying could be an indirect effect. For instance, the presence of Ovipostatin could result in higher paternity by increasing storage of the donated sperm and/or displacing already-stored rival sperm. Alternatively, by postponing egg laying in the partner the latter may be committed to invest more resources per egg, thus increasing egg quality (rather than quantity). Clearly, further research is needed to tease these different explanations apart and the developed intravaginal injection approach will be indispensable for this. In addition, the exact mode of action of Ovipostatin needs to be unraveled further, especially in light of the finding that ejaculate receipt coincides with an inhibition of the central neurons that control egg laying behavior in this species [32], [34].

Interestingly, the reduction in egg laying might cause a conflict of interest within these hermaphrodites. For one thing, without Ovipostatin the recipient would have produced more offspring. Also, other examples exist where the reproductive success of the male occurs at the expense of female fitness [7], [9], [36]. Hence, it is possible that in this hermaphrodite the male and female interests within a mating interaction do not coincide. Evidently, whether this really represents a sexual conflict remains to be shown for this system. So far, such sexual conflicts have been demonstrated most convincingly for hermaphrodites with extreme and unusual reproductive habits, usually involving stabbing devices that transfer sperm or allohormones [24], [37]. The present study shows that, in hermaphrodites that simply transfer seminal substances along with the sperm during insemination, rather than by using a stabbing device, sexual selection and conflict may be of equal importance. Evidently, if the sexual roles are indeed in conflict here, this also provides scope for investigating the possibility of the recipient to evolve resistance traits to counteract the effect of a specific seminal fluid component (persistence trait). Such antagonistic co-evolution could then be the driving force behind the evolution of hermaphrodites' ejaculates towards complex mixtures of sperm and numerous seminal fluid components [as has been shown to occur in insects, 38].

To conclude, sexual selection research on hermaphrodites seems to be biased towards mating behaviors involving stabbing devices that transfer sperm or accessory gland substances. Here, we demonstrate that the simple transfer of accessory gland substances along with sperm also occurs. It is likely that this is much more common in internally-fertilizing hermaphrodites than currently reflected in the literature. Moreover, since hermaphrodites express genes for male *and* female reproductive pathways, active compounds for partner manipulation are more easily available and only require appropriate means for delivery [23], [39]. The prediction would then be that such allohormones evolve more easily in hermaphrodites, in comparison with separate sex species, and may show more resemblance with already existing regulatory peptides.

## Methods

### Animals

The animal work has been conducted according to national and international guidelines. All specimens of *Lymnaea stagnalis* (L.) were obtained from the laboratory culture of the VU University. In the breeding facility and experimental tank the low-copper water was kept at 20°C and the light:dark cycle was 12 h:12 h. Non-virgin adult snails were sexually isolated in perforated plastic jars (460 ml) within the experimental tanks, which had continuous water exchange. Snails were each fed a circular disc (19.6 cm^2^) of lettuce per day, which was slightly below their maximum daily food intake and therefore completely consumed.

For histology, the prostate gland was removed from a mature individual that had been injected with 2–3 ml of 50 mM MgCl_2_ through the foot into the haemocoel. The prostate gland was then immediately fixed in Bouin's solution O/N and subsequently transferred to 70% ethanol. After dehydration in ascending alcohol concentrations, the prostate gland was embedded in paraffin for serial sectioning at 7 µm. The sections were stained with both azan and haematoxylin and eosin.

### Peptide and protein extraction and purification

Peptides and proteins were prepurified from extracts of prostate glands in six separate batches by using solid phase extraction. Prostate glands were removed from 243 adult specimens that had been sexually isolated for two weeks and had a shell length between 26 and 30 mm. These glands were lyophilized, extracted at 4°C in 0.1% heptafluorobutyric acid (HFBA; Pierce, Rockford IL) using a Polytron homogenizer (Brinkmann Instruments, Inc., Westbury, NY), and sonicated. The extract was centrifuged for 20 min at 20,000×*g* (4°C), and the supernatant was purified on C18 Sep-Pak Vac cartridges (5 g; Waters Associates, Milford, MA); Sep-Pak Vac cartridges were pre-treated with 5 ml of 100% acetonitrile (ACN) containing 0.1% HFBA and rinsed with 10 ml of 0.1% HFBA. The peptides and proteins were eluted with 15 ml of 70% ACN containing 0.1% HFBA and lyophilized. The lyophilizate was resuspended in 0.1% HFBA and applied to a semi-preparative C18 reversed-phase (RP)-HPLC column (10×250 mm; Vydac, Hesperia, CA). The column was eluted with a two-step linear gradient of 0.1% HFBA and ACN containing 0.1% HFBA. The first step was 0–10% ACN containing 0.1% HFBA in 5 min, and the second step was 10–58% ACN containing 0.1% HFBA in 170 min. The column eluate was monitored at 215 nm, and 1 min (1 ml) fractions were collected. For chemical characterization studies and bioassays, fractions containing peaks of interest were pooled, lyophilized, and repurified by Vydac C18 RP-HPLC using a two-step linear gradient of 0.1% trifluoroacetic acid (TFA; Pierce) and ACN containing 0.1% TFA. The first step was 0% ACN containing 0.1% TFA for 5 min, and the second step was 0–60% ACN containing 0.1% TFA in 200 min; 1 min (1 ml) fractions were collected. The ten most prominent peaks were characterized by amino acid microsequence analysis and those containing peptides or proteins were used for the bioassay.

### Transferred amounts

Before microsequencing and peptide/protein testing, we verified whether the peaks of interest were indeed transferred substances, and not components of the prostate gland tissue itself. Therefore, we compared peptide and protein profiles of intact prostate glands with those of seminal fluid (expressed prostate gland contents gently squeezed out of the lumen) and prostate gland tissue (remaining gland tissue after expressing contents). This confirmed that the peaks of interest were indeed peptide and protein components of the seminal fluid. For this test we used a total of 30 snails with a shell length of 26 to 30 mm that had been isolated for two weeks.

To determine the amount of sperm and seminal fluid transferred in a single insemination, we isolated animals (26 to 30 mm) for two weeks and allowed one subset to inseminate a partner once (observed during 5 h), while keeping the other subset isolated. After this copulation we dissected out the seminal vesicles and prostate gland of the sperm donors as well as the subset that remained in isolation. The change in weight of the seminal vesicles provides an indication of the amount of sperm transferred during one insemination, while the change in prostate gland weight indicates seminal fluid transferred. The seminal vesicles weighed, respectively, 11.09±4.77 mg (N = 12) and 7.48±3.47 mg (N = 18). Thus, the seminal vesicles are approximately 33% lighter after sperm donation, which represents a significant difference (Wilcoxon χ^2^ = 3.96, df = 1, p = 0.047), and since the tissue of this organ is extremely thin this provides a proper estimate of the weight of the sperm transferred in a single insemination. The prostate glands weighed, respectively, 52.88±9.36 mg (N = 14) and 43.84±10.48 mg (N = 19), indicating that the prostate gland is approximately 17% lighter after seminal fluid donation as compared to before, i.e. in the isolated controls (Wilcoxon χ^2^ = 5.78, df = 1, p = 0.016). Since the tissue of the prostate gland represents about half of the weight of the organ, we estimated that the transferred amount represents between 25 and 33% of the seminal fluid stored in the gland. For the peptide and protein test we therefore used one third of the content of the seminal vesicles and the prostate gland products as the intravaginal injection dose per individual.

### Microsequence analysis

For Edman degradations, samples were applied to Biobrene Plus-treated glass fiber filters and subjected to automated N-terminal sequence analysis using a PE/Applied Biosystems Procise 494/HT Protein Sequencer. Pulsed liquid cycles were used for each analysis. Data analyses were conducted by direct inspection on on-line analog chart recordings and compared with phenylthiohydantion-amino acid standards.

### Bioassays

In order to test the effect of the seminal fluid peptides and proteins on recipients we performed the following experiments. In total we tested the effect of 8 peptides or proteins and ran one control experiment using a total of 368 snails with a mean size of 29.2 mm (SD: ±3.2). Each peptide or protein was tested separately, with each treatment and control initially containing 16 individuals. For practical reasons, the peptide and protein tests were separated into 4 experiments using 80 snails each. In each of these experiments 2 peptides/proteins were tested separately, both with and without sperm, and included a carrier medium control (physiological saline), thus resulting in 5 treatments per test. See Table 1 for details about the testing order. We performed one additional control experiment, using 48 snails, to exclude effects of traces of the HPLC solvent (HFBA) or sperm alone in saline, again with the carrier medium as control. Since the effect of the complete seminal fluid has already been tested in a previous study using exactly the same method (Koene et al. 2009), we did not include this treatment here again.

At the start of each experiment, two of the freeze dried fractions, each containing one of the eight peptides/proteins of interest, were briefly centrifuged and then resuspended in filtered (0.2 µm) saline. For each peptide or protein, an amount sufficient for inseminating 32 animals (two times 16) with 0.33 prostate gland equivalents each was aliquoted and put on ice. Each peptide/protein aliquot was further divided in two in order to test the peptides/proteins both with and without sperm (N = 16 for each). Sperm were obtained from donor snails. These were taken from the breeding facility and had been isolated for one week and fed lettuce *ad libitum* to allow for autosperm replenishment [40]. Each donor snail was anaesthetized by means of an injection of approximately 2 ml of 50 mM MgCl_2_ through the foot. The seminal vesicles were then dissected out, placed on ice in a collection vial and suspended in saline [28]. The suspended sperm were then immediately added to the test solutions (for treating one time 16 animals per peptide or protein) at a concentration of 0.33 seminal vesicle equivalent (see above). A control containing saline only was prepared at the same time.

For intravaginal injection, 1-ml syringes were filled with the different solutions and each syringe was fitted with a blunted injection needle. A silicon tube with a diameter of 1 mm and a length of 10 cm, was slid over each injection needle. The randomly chosen experimental animals were inseminated intravaginally with either the control or one of the test solutions in an alternating fashion. Prior to intravaginal injection, each snail was anaesthetized with 2 ml of 50 mM MgCl_2_. This anaesthetic ensured that the animal was completely relaxed and maximally extended from its shell within seconds. The silicon tube was then carefully inserted into the female gonopore, now clearly visible as a white spot on the right side of the animal anterior to the right tentacle and male gonopore. A volume of 0.03 ml was then slowly injected after which the silicone tube was pulled out. Following this procedure, the shell length of each injected snail was measured and the snail was returned to its perforated plastic jar in the large experimental tank, where it would recover over the next hours.

Following intravaginal injection, the animals were kept in their individual perforated plastic jars throughout the experiment, which lasted 12 days. Every day each snail was provided with an excess of lettuce discs each measuring 19.6 cm^2^. During the experiment we monitored body size, egg laying, hatching success and food consumption. Body size was measured both as length of the shell and wet body weight. We measured the number of egg masses produced and the number of eggs per egg mass. For hatching success, counted egg masses were kept in separate petri dishes filled with low-copper water that was refreshed twice a week and were left to hatch over the next three weeks. Once all the developing eggs had hatched, the unhatched eggs were counted and the hatching success (percentage of hatched eggs) was calculated. Consumption of the provided lettuce was quantified daily per snail by collecting the lettuce remains from its jar and taking a digital picture. The digital camera was placed at a standardized distance. The surface of the remains of the lettuce discs was measured using ImageJ [NIH, 41]. Consumption was calculated by subtracting the leftover area from the total area of the lettuce provided [34], [42]. Previous work has already shown that lettuce surface area can be used as a reliable measure for nutritional intake [42].

Statistical analyses were performed with JMP version 5.0.1 (SAS Institute Inc.). The data were tested for normality and nonparametric methods were used wherever necessary.

### Molecular analysis of the Ovipostatin gene

For this and the next section we used 20 individuals of 30 mm-shell length that had mated and were subsequently sexually isolated for one week. From these animals, we dissected out the albumen gland (AG), the central nervous system (CNS), foot tissue, the penial complex (Pe), the prostate gland (PG) and the seminal vesicles (SV).

Based on the N-terminal Ovipostatin sequence, 3′-RACE was used to determine the remaining cDNA sequence. Total RNA was isolated from the prostate gland using the TriReagent (Molecular Research Centre), following the manufacturer's instructions and first-strand cDNA was generated by RT using antisense adaptor primer (5′-AAGCAGTGGTATCAACGCAGAGT_17_-3′) and the Superscript Preamplification System for First Strand Synthesis (Invitrogen).

3′-RACE was performed using a degenerate sense primer corresponding to the Ovipostatin sequence VSPDTFE (5′-NGTNWSNCCNGAYACNTTYGARGC-3′) and a 3′-RACE antisense primer (5′-AAGCAGTGGTATCAACGCAGAGT-3′). PCR reactions had a final concentration of 1x PCR Buffer, 1.5 mM MgCl_2_, 200 µM dNTPs, 2.0 µM of sense and antisense primer, and 0.25 units of Red*Taq* polymerase (Sigma). Samples were heated at 94°C for 2 min and amplified for 36 cycles (94°C, 1 min; 45°C, 1 min; 72°C, 2 min), followed by a 7-min extension at 72°C. Semi-nested PCR was performed with a second sense primer (ANLYGTDG; 5′-RGCNAAYYTNTAYGGNACNGAYGG-3′) and the same 3′-RACE antisense primer. Samples were heated at 94°C for 2 min and amplified for 36 cycles (94°C, 30 sec; 48°C, 1 min; 72°C, 1 min), followed by a 7-min extension at 72°C. RACE products were cloned into a pGEM-T vector (Promega), sequenced, and consensus sequences created in Sequencher*t* 3.1.1 (Gene Codes Corporation).

5′-RACE was performed with primers designed from 3′-RACE sequences and using the SMART-RACE kit (BD Biosciences) according to the manufacturer's instructions. Primer sequences are available upon request.

### Tissue expression of Ovipostatin by RT-PCR

RT-PCR of *L. stagnalis* albumen gland, pooled central nervous system, foot tissue, penial complex, prostate gland and seminal vesicle RNA was performed to determine tissue expression of their corresponding mRNAs. Total RNA was isolated using TriReagent (Molecular Research Centre). RNA quantity and quality was assessed using UV spectrophotometry (NanoDrop ND-1000) and agarose gel electrophoresis. First-strand cDNA was generated by reverse transcription of 1 µg total RNA using an antisense adaptor primer and the Superscript Preamplification System for First Strand Synthesis. PCR was performed using the primer combinations: sense, 5′-TGTGTGCGGAAAGTATGCTGTC-3′ and antisense 3′-RACE AAGCAGTGGTATCAACGCAGAGT. As a negative control, the PCR reaction contained no cDNA. PCR reactions had a final concentration of 1x PCR Buffer, 1.5 mM MgCl_2_, 200 µM dNTPs, 0.5 µM of sense and antisense primer, and 0.25 units of Red*Taq* polymerase (Sigma). Samples were heated at 94°C for 2 min and amplified for 36 cycles (94°C, 30 sec; 50°C, 1 min; 72°C, 1 min), followed by a 7-min extension at 72°C. Reactions were fractionated on a 2% agarose gel, stained with ethidium bromide and visualized.

## Supporting Information

Table S1 Amino acid sequences of the major HPLC peaks found in the prostate gland of Lymnaea stagnalis
(0.03 MB DOC)

Table S2 Effects of eight Lymnaea stagnalis seminal fluid peptide and protein peaks
(0.08 MB DOC)
