# Supplementary material for: Male Accessory Gland Protein Reduces Egg Laying in a Simultaneous Hermaphrodite
Source: PLoS One. 2010 Apr 12;5(4):e10117. doi: 10.1371/journal.pone.0010117 (PMC2853560; doi:10.1371/journal.pone.0010117)
Supplement: Table S1 — Amino acid sequences of the major HPLC peaks found in the prostate gland of Lymnaea stagnalis (0.03 MB DOC) [file pone.0010117.s001.doc]

**Table S1. Amino acid sequences of the major HPLC peaks found in the prostate gland of *Lymnaea stagnalis*.**

| **Peak** | **Amino acid sequence** | **Residues** |
| --- | --- | --- |
| 3 | + - + - --+  APGSI**C**PRP**P**EIKGDS**DD**R**P**P | 21 |
| 4 | -- - + +  LDDGESNGQSHIKATIPK | 18 |
| 5 | -- - +-+ -+ - - + + -  SPADENDPSKEKLNAFGGADKLLEQIDALGPPMKVKPSGSE | 41 |
| 7a | +- + ++ +--- + + + + - - - +  GKDSPSKVXIKKXIGTFAKDDDXSKYFKXKGVKPDYYSXYXGQLFDADXKMXI… | >53 |
| 7b | - - - +- -++ - +- +  YCDTGDLDPREMSLGQFSLAFDKKGICNECNCNRDTGLSCCQCSPR… | >46 |
| 8a | +- + - - + + + ++-+ + - ---+  GKDPKIIENEHKAKLKLRKDKLKQDTSQMDDEKPFXNSXXF… | >41 |
| 8b | -- --- - -- --- - - + - ++ + -  DEGDDDNTETDDYGTGPITYNFPADDETDXFFVEWRMFXDAXWKRYNQAAGRXLD… | >55 |
| 10 | -+- - - - + -  EKDQTPSXSPDTFEANLYGTDGSVXGKYAVD… | >31 |

Residues indicated in bold in Peak 3 are predicted based on the mass of the secondary peptide sequence. Unresolved residues are indicated by an X. Peaks 7a, 7b, 8a, 8b, and 10 are partial sequences. The locations of charged residues in the peptides and proteins are indicated by + and -. The protein Ovipostatin corresponds to Peak number 10.
