# Supplementary material for: Male Accessory Gland Protein Reduces Egg Laying in a Simultaneous Hermaphrodite
Source: PLoS One. 2010 Apr 12;5(4):e10117. doi: 10.1371/journal.pone.0010117 (PMC2853560; doi:10.1371/journal.pone.0010117)
Supplement: Table S2 — Effects of eight Lymnaea stagnalis seminal fluid peptide and protein peaks (0.08 MB DOC) [file pone.0010117.s002.doc]

**Table S2. Effects of eight *Lymnaea stagnalis* seminal fluid peptide and protein peaks.**

| **Test** | **Treatments** | **Body size** | | | **Egg mass production** | | | **Hatching** | | | **Consumption** | | |
| --- | --- | --- | --- | --- | --- | --- | --- | --- | --- | --- | --- | --- | --- |
|  |  | *N* | *Mean* | *SD* | *N* | *Mean* | *SD* | *N* | *Mean* | *SD* | *N* | *Mean* | *SD* |
| **A** | Peak 3 | 13 | 30.85 | 1.99 | 13 | 0.85 | 0.69 | 9 | 89.90 | 4.01 | 13 | 46.77 | 39.42 |
|  | Peak 3 + sperm | 16 | 30.81 | 2.01 | 16 | 1.31 | 1.01 | 12 | 92.19 | 2.98 | 16 | 36.75 | 73.83 |
|  | Peak 4 | 15 | 30.20 | 1.52 | 15 | 0.47 | 0.74 | 5 | 91.45 | 4.58 | 15 | 38.40 | 35.31 |
|  | Peak 4 + sperm | 14 | 31.14 | 2.41 | 14 | 0.79 | 0.70 | 9 | 89.97 | 7.34 | 14 | 46.29 | 45.98 |
|  | Control | 16 | 30.56 | 1.90 | 16 | 1.06 | 0.93 | 11 | 90.92 | 3.09 | 16 | 37.56 | 46.28 |
| **B** | Peak 7a | 15 | 26.27 | 2.22 | 16 | 0.87 | 0.72 | 11 | 93.54 | 2.94 | 16 | 16.19 | 25.44 |
|  | Peak 7a + sperm | 14 | 26.14 | 1.70 | 15 | 0.93 | 0.80 | 10 | 93.40 | 2.46 | 15 | 20.67 | 22.29 |
|  | Peak 7b | 16 | 27.12 | 2.12 | 16 | 1.00 | 1.03 | 10 | 93.50 | 2.27 | 16 | 21.31 | 21.42 |
|  | Peak 7b + sperm | 13 | 25.61 | 1.98 | 13 | 0.69 | 0.63 | 8 | 93.50 | 2.98 | 13 | 19.92 | 17.33 |
|  | Control | 14 | 26.57 | 2.21 | 15 | 0.93 | 0.88 | 10 | 94.80 | 2.74 | 15 | 26.53 | 21.52 |
| **C** | Peak 8a | 16 | 28.88 | 2.19 | 16 | 0.94 | 0.68 | 12 | 91.75 | 6.72 | 16 | 31.75 | 36.80 |
|  | Peak 8a + sperm | 12 | 29.42 | 2.07 | 12 | 0.50 | 0.52 | 5 | 91.40 | 1.95 | 12 | 14.75 | 24.49 |
|  | Peak 8b | 16 | 30.00 | 2.31 | 16 | 1.06 | 0.57 | 14 | 91.71 | 4.21 | 16 | 34.19 | 45.61 |
|  | Peak 8b + sperm | 16 | 29.13 | 2.00 | 16 | 0.81 | 0.66 | 11 | 93.36 | 3.14 | 16 | 12.25 | 14.99 |
|  | Control | 13 | 29.85 | 3.36 | 13 | 1.08 | 0.64 | 10 | 94.00 | 3.62 | 13 | 18.00 | 25.36 |
| **D** | Peak 5 | 16 | 30.19 | 2.69 | 16 | 1.25 | 0.68 | 15 | 92.62 | 5.44 | 16 | 42.00 | 52.89 |
|  | Peak 5 + sperm | 16 | 30.25 | 2.54 | 15 | 1.13 | 0.74 | 13 | 93.02 | 2.29 | 16 | 31.88 | 38.43 |
|  | Peak 10 | 15 | 31.40 | 2.29 | 15 | 0.73 | 0.59 | 10 | 92.28 | 2.46 | 15 | 43.13 | 37.00 |
|  | Peak 10 + sperm | 15 | 31.20 | 2.24 | 15 | 0.67 | 0.62 | 9 | 92.32 | 3.24 | 15 | 39.07 | 46.81 |
|  | Control | 15 | 30.67 | 2.61 | 15 | 1.13 | 0.35 | 15 | 94.31 | 4.31 | 15 | 54.40 | 61.52 |
| **E** | HFBA | 19 | 29.47 | 2.97 | 19 | 0.36 | 0.31 | 12 | 0.72 | 0.17 | 19 | 43.79 | 56.27 |
|  | Sperm | 19 | 30.37 | 1.95 | 19 | 0.50 | 0.39 | 14 | 0.80 | 0.07 | 19 | 29.42 | 46.85 |
|  | Control | 19 | 30.16 | 3.04 | 21 | 0.29 | 0.32 | 12 | 0.72 | 0.23 | 21 | 33.95 | 46.16 |

The number of animals (N), the means and standard deviations (SD) are shown per treatment for their body size (shell length in mm), egg mass production (total number of masses laid), hatching success (percentage of hatched eggs) and consumption (in cm^2^ lettuce). The separate experiments, done at different dates, are indicated by the letters A to E. The plus sign indicates that the peptide or protein peak was tested with sperm (+ sperm). The protein Ovipostatin corresponds to Peak number 10. The results of statistical testing are shown in Table 1.
